# Research Conducted Using Data Obtained through Online Communities: Ethical Implications of Methodological Limitations

**DOI:** 10.1371/journal.pmed.1001328

**Published:** 2012-10-23

**Authors:** A. Cecile J. W. Janssens, Peter Kraft

**Affiliations:** 1 Department of Epidemiology, Erasmus University Medical Center Rotterdam, The Netherlands; 2 Department of Epidemiology, Rollins School of Public Health, Emory University, Atlanta, Georgia, United States of America; 3 Program in Molecular and Genetic Epidemiology, Harvard School of Public Health, Boston, Massachusetts, United States of America

## Abstract

An Essay by A. Cecile Janssens and Peter Kraft discusses the limitations inherent in research involving collection of self-reported data by self-selected participants, and makes proposals for upfront communication of such limitations to study participants.

**Summary Points**

- An increasing number of public/private initiatives are exploring novel ways of conducting scientific research, including the use of social media and online collection of self-reported data.
- Research relying on collection of self-reported data by self-selected participants has known methodological limitations, including selection bias, information bias, and confounding.
- Such limitations may mean that results and conclusions of research using data obtained through online communities need to be interpreted with caution, as further replication is often required.
- The findings of research, including their potential actionability, should be communicated to participants in a way that is understandable, accurate, complete, and not misleading.
- The potential for sharing participants' data with third parties as well as the commercial uses of research findings should be disclosed to participants prior to consent.

*This is one article in an occasional* PLOS Medicine *series on research integrity that examines issues affecting the ethics of health research worldwide.*

## Introduction

An increasing number of public and commercial initiatives invite individuals to participate in scientific research via the internet (Table 1). People are asked to provide information about personal medical history, medications, physical traits and measurements, ethnicity/ancestry, lifestyle and environmental exposures, and to donate biological material, generally saliva or blood, for DNA analysis. Some initiatives, such as the Personal Genome Project, have been launched with the specific goal of conducting scientific research, whereas others perform scientific analyses using data that were at least partly collected for other purposes. For example, PatientsLikeMe is an online community where patients can share information on symptoms, health state, and treatments to learn from each others' experiences, and the company 23andMe sells personal genome tests to individuals who want to learn their genetic risks of common diseases, carrier status of rare diseases, response to drug treatment, and ancestry. Data are collected predominantly through self-report online questionnaires and some initiatives offer the opportunity to make data accessible for the public. For example, the Personal Genome Project publishes anonymized data online and participants of PatientsLikeMe can choose to publish all data publicly available on the web or make data accessible only to registered users.

**Table 1:** Examples of online research initiatives.

Initiative	Aims and Claims
PatientsLikeMe.org	“To provide a better, more effective way for you to share your real-world health experiences in order to help yourself, other patients like you and organizations that focus on your conditions.”
23andMe.com	“Our research arm, 23andWe, gives customers the opportunity to leverage their data by contributing it to studies of genetics. With enough data, we believe 23andWe can produce revolutionary findings that will benefit us all.”
Personal Genome Project (personalgenomes.org)	“The mission of the Personal Genome Project is to encourage the development of personal genomics technology and practices that: are effective, informative, and responsible; yield identifiable and improvable benefits at manageable levels of risk; are broadly available for the good of the general public.”
DIYgenomics.com	“A non-profit research organization founded in March 2010 to realize personalized medicine through crowdsourced health studies and apps.”
Genomera.com^a^	“We're crowd-sourcing health discovery by helping anyone create group health studies.”
Curetogether.com^b^	“Bringing patients into research as active partners is one of our big missions at CureTogether.” [21]

Quoted information was downloaded from the organizations' websites on July 1, 2012.

a Beta version.

b Acquired by 23andMe.

Strong claims regarding the benefits of research using these resources are often made in order to encourage individuals to provide personal (health) information. For example, 23andWe, the research arm of 23andMe “gives customers the opportunity to leverage their data by contributing it to studies of genetics. With enough data, we believe 23andWe can produce revolutionary findings that will benefit us all” [1]. PatientsLikeMe tells patients that sharing personal stories and health data does not only enable individuals to “put your disease experiences in context and find answers to the questions you have” but also gives “the opportunity to help uncover great ideas and new knowledge” [2]. But how valid are these claims? Can online data collection lead to major breakthroughs in health research? We worry that overstating the conclusions that can be drawn from these resources may impinge on individual autonomy and informed consent. Just as researchers must take care to accurately convey direct benefits to study participants (which, we argue, in these situations are often small), they should also describe the likely outcomes and known limitations of observational studies conducted using volunteers. Clarity regarding the benefits of research using solicited personal data is particularly important when the data collected are also used for other purposes (e.g., PatientsLikeMe may sell members' information to pharmaceutical and insurance companies [2]), lest the allure of participation in a scientific study be used as a Trojan horse to entice individuals to part with information they might not otherwise volunteer.

## “Revolutionary” Findings?

As early examples of such initiatives, 23andMe and PatientsLikeMe have already published their first scientific results. Using self-reported phenotypic data provided by their customers, 23andMe reported that they replicated over 180 genetic associations from the catalogue of genomewide association studies (GWAS) of the National Human Genome Research Institute's Office of Population Genomics [3], identified genetic associations for miscellaneous traits long suspected of having a genetic basis [4], and identified two novel loci and a substantial genetic component for Parkinson disease [5]. And in a study of 447 patients, PatientsLikeMe showed that lithium carbonate did not affect the rate of progression in amyotrophic lateral sclerosis (ALS) [6].

But how valid and new are these findings? One of the loci for Parkinson disease that 23andme discovered was confirmed in collaboration with the International Parkinson Disease Genomics Consortium [7], but the other loci need further replication [8],[9], and also the newly identified associations for various traits still need to be replicated in independent samples [4]. The replication of 180 associations concerned 144 out of 392 attempted associations in case-control and quantitative phenotypes from the GWAS catalogue and 39 out of 106 attempted associations with phenotypes that were in weak correspondence with those in the catalogue. In both instances, the observed percentage of replications was less than expected based on the statistical power for each of the phenotypes tested [3]. And finally, as acknowledged by the authors, the absence of an association between lithium carbonate and ALS progression reported by PatientsLikeMe was in line with earlier observations and two prematurely stopped randomized clinical trials [10],[11]. Still, it is not clear that the absence of a statistically significant finding of this particular study can be interpreted as the absence of a treatment effect, given the methodological limitations in online data collection. Using self-reported data from self-selected individuals is subject to several known biases in the presence of which reported frequencies, prevalences, and associations can be over- or underestimated. Table 2 lists the sources of bias in observational studies that are commonly observed but particularly relevant for studies using self-reported data from self-selected individuals [12]: selection bias, information bias, and confounding.

**Table 2:** Biases in observational studies and their potential effect when using self-report data from self-selected individuals [12].

Bias	Problem When:
Selection bias	Bias occurring in the selection of the population: population studied is not representative for target population
Ascertainment bias	Inappropriate definition of the eligible population
Non-participation bias	Non-participation is related to the outcome or risk factors investigated, e.g., depression
Healthy volunteer bias	Participants are healthier than general or target population
Information bias	Bias occurring during data collection: systematic measurement error
Misclassification bias	Imperfections in procedure to classify exposures or disease status
Detection bias	Presence of risk factors increases probability that disease is diagnosed
Recall bias	Recall of risk factors differs between individuals patients and nonpatients
Reporting bias	Reporting of risk factors differs between patients and nonpatients, e.g., patients with lung cancer may underreport smoking status
Hawthorne effect	Awareness of being observed influences outcome of the study, e.g., participants complete exposure/disease status on the basis of observed associations
Confounding	Observed risk factor is correlated with unmeasured risk factor
By indication	Prognostic factors influence treatment decisions

## Sources and Implications of Bias

The first source of bias, selection bias, occurs when the study population does not represent the target or sampling population, for example when customers of personal genome tests are healthier, higher educated than the general population [13], or when participating patients are more motivated, literate, and empowered [14],[15]. Selection bias is also observed when participation in a study by cases is related to a certain risk factor and participation amongst control individuals is unrelated to that factor, e.g., when depressed people are less likely to join online communities. In that example, the validity of studies in psychiatric, neurological, and geriatric diseases might be reduced, because the frequency of the risk factor in cases and its impact on disease risk are likely underestimated. Statistical techniques, such as inverse-probability sample weighting, can correct the effects of selection bias, but these require that the sampling population is known. The fact that the sampled population is unknown is a major shortcoming in studies that recruit online through participant self-selection.

The second source of bias, information bias, concerns any systematic error in the collection of data. Errors in exposure reporting that are unrelated to the phenotype being studied (“non-differential misclassification”) cannot create an association when none truly exists, although they can attenuate the estimated size of a true association. Of greater concern, errors that are related to the phenotype being studied (“differential misclassification”) can create spurious associations where none exist, or over- or underestimate the size of true associations. For example, individuals with a disease may recall their exposure history differently than those without (reporting and recall biases), especially if the exposure is widely suspected to be linked to the disease.

Misclassification of outcome typically occurs for outcomes that apparently follow from certain exposures (detection bias). In studies with continuous online data collection, outcome misclassification may be particularly troublesome because participants may report their phenotype status after learning about their risk factors and their impact on the phenotype [4],[16]. 23andMe suspected this source of bias for several traits, including athletic performance [4]. They observed that self-report of athletic status, i.e., performance in sprint or endurance races, was more in line with the observed genotype-phenotype association among customers who had viewed their genotype status prior to completing the questionnaire. Information bias might also be a problem when openness of data is encouraged such as with PatientsLikeMe. Patients can view risk factors and symptoms of other individuals before they complete their questionnaire, which may lead to biased representation of the clustering of symptoms. In general, self-reported data are known to be subject to misclassification of outcome because lay people are less aware of formal definitions and diagnostic criteria. Misclassification in the outcome variable is a serious concern, particularly when epidemiological associations are expected to be small, such as is in genetic studies in multifactorial diseases.

The third source of bias, confounding, occurs when two variables are associated because both are associated to a third that might explain the association between the two. Confounding can be effectively dealt with using stratified or multivariable regression analyses when the confounding variables are measured. An advantage of online data collection is that additional questions can be asked of participants, but there are sources of confounding that cannot be solved this way. It is difficult to reliably assess confounders retrospectively and to correct bias that is caused by confounders that affect the probability of participation. Examples of potential confounders that may be associated with the probability of participation in online studies are socio-economic status and health literacy.

## Opportunities for Research

While these biases can greatly affect the interpretation and generalizability of what can be done with self-reported data collected from volunteers, there are many situations where these data may prove useful. First, analyses can be done on risk factors and outcomes that are less susceptible to misclassification because the phenotype definition or methods of assessment are straightforward, such as for demographic information and for diseases that less likely remain undiagnosed, e.g., cancer, Parkinson disease, and ALS. Second, the data can be used for analyses where the selection of individuals is the preferred study design. Many gene discovery studies are performed using so-called extreme group comparisons, i.e., comparing patients with screened controls or comparing patients with a family history of disease with unscreened controls. Screening controls on the absence of any symptoms related to the disease of interest may compensate for potential misclassification of the outcome. And third, the data can still be used in analyses for which the presence of bias does not affect the conclusion of the study—analyses where bias may affect the magnitude of association, but not the presence of association. When expected associations are large or when the sample size is large, associations may still be significant in the presence of misclassification. But other than these, the opportunities for research are limited, as the results obtained using self-reported data from self-selected individuals may not easily withstand skepticism about the biased approach.

## Concluding Remarks

The new initiatives of public participation in science (citizen science) by online and continuous data collection have changed our views on how to most efficiently and effectively conduct scientific studies [17], and their greatest value may be in that area. These initiatives can speed up scientific research by facilitating the recruitment of participants in a relatively easy way, which is particularly relevant for rare diseases such as ALS and Parkinson disease. PatientsLikeMe has a trial search tool, linked to clinicaltrials.gov, through which patients can see which trials are still recruiting [2]. And with their rich data collections and online opportunities for fast data updates, they can quickly put new topics on the scientific agenda and question published observational studies and trials. An excellent example was provided by 23andMe. Within a week of the high-profile publication of a putative genetic predictor of longevity, 23andMe showed that the predictor did not replicate in their data. After re-examination of their study protocol and data analysis, the authors of the longevity study retracted their initial publication [18],[19]. Nevertheless, the biases in the design and data collection of the citizen science organizations warrant that most conclusions from their studies need further replication.

Initiators of online data collections are strong advocates of openness and transparency, but they are relatively reserved about the methodological limitations of their research in communications to their participants. Their scientific papers acknowledged and extensively discussed the limitations of their study designs and data collection, including the need for replication of the findings and the need for further research [3]–[6],[20], but this concern is not necessarily reflected on their websites, where they encourage people to provide information and where they list and describe their scientific discoveries. Presenting results without a proper explanation and disclaimer is however not without risks. When PatientsLikeMe reports that lithium does not reduce ALS progression, will patients discontinue treatment? Will they still trust their doctors when they were prescribed a drug that apparently does not work? Researchers should clearly explain the limitations of their approach and their findings and stress that participants should not change their medical regimens without consultation of their doctor (Table 3).

**Table 3:** Recommendations for communicating opportunities and limitations of research conducted using data obtained through online communities.

Timeline	Recommendations and/or Limitations
Before data collection:	Information about what can and cannot be done with the data collected
	Clear discussion of immediate benefits that study participants may or may not receive
	Presentation of realistic and fair claims about scientific knowledge that is likely to be gained
	Disclosure about potential for sharing participants' data with third parties as well as the commercial uses of research findings
After data analyses:	Comprehensive and balanced presentation of research results
	Clear interpretation of results, especially in light of other studies and need for replication
	Discussion of implications for health behavior or medical decisions, if any

We have focused on the ethical implications of methodological limitations of research involving self-reported data from self-selected participants. Research using data obtained through online communities faces new dilemmas in relation to old issues, which require further ethical analysis and public debate, including the provision of adequate consent, the safeguard of public trust, disclosure of commercial development of research results, and the sale of participants' data to third parties [17]. Only a responsible approach with realistic expectations about what can be done with and concluded from the data will benefit science in the long run.
